# Respiratory syncytial virus modifies microRNAs regulating host genes that affect virus replication

**DOI:** 10.1099/vir.0.044255-0

**Published:** 2012-11

**Authors:** Abhijeet Bakre, Patricia Mitchell, Jonathan K. Coleman, Les P. Jones, Geraldine Saavedra, Michael Teng, S. Mark Tompkins, Ralph A. Tripp

**Affiliations:** 1 Department of Infectious Diseases, University of Georgia, Athens, GA 30602, USA; 2 Division of Allergy and Immunology, Department of Internal Medicine, USF Health, Tampa, FL 33612, USA

## Abstract

Respiratory syncytial virus (RSV) causes substantial morbidity and life-threatening lower respiratory tract disease in infants, young children and the elderly. Understanding the host response to RSV infection is critical for developing disease-intervention approaches. The role of microRNAs (miRNAs) in post-transcriptional regulation of host genes responding to RSV infection is not well understood. In this study, it was shown that RSV infection of a human alveolar epithelial cell line (A549) induced five miRNAs (let-7f, miR-24, miR-337-3p, miR-26b and miR-520a-5p) and repressed two miRNAs (miR-198 and miR-595), and showed that RSV G protein triggered let-7f expression. Luciferase–untranslated region reporters and miRNA mimics and inhibitors validated the predicted targets, which included cell-cycle genes (*CCND1*, *DYRK2* and *ELF4*), a chemokine gene (*CCL7*) and the suppressor of cytokine signalling 3 gene (*SOCS3*). Modulating let-7 family miRNA levels with miRNA mimics and inhibitors affected RSV replication, indicating that RSV modulates host miRNA expression to affect the outcome of the antiviral host response, and this was mediated in part through RSV G protein expression.

## Introduction

Respiratory syncytial virus (RSV) is an important paediatric and geriatric challenge causing substantial hospitalizations, clinic visits and >14 000 deaths per annum (CDCP, 2008). RSV is a prototype of the genus *Paramyxovirus* with a 15 kb negative-sense ssRNA genome encoding 11 proteins (NS1, NS2, N, P, M, SH, G, F, M2-1, M2-2 and L). Despite 60 years of intense efforts towards an RSV vaccine, there is a lack of effective prophylactic and therapeutic intervention, mainly due to a poor understanding of the host–virus interface. Whilst recent antiviral efforts have begun to target host pathways to inhibit virus replication (Li *et al.*, 2009; Meliopoulos *et al.*, 2012; Panda *et al.*, 2011) and RNA interference approaches using small interfering RNAs (siRNAs) to target RSV have shown success at reducing virus replication in a mouse model (Alvarez *et al.*, 2009) and are currently in phase II clinical trials (Alvarez *et al.*, 2009), mitigating the host immune response that results in bronchiolitis remains a challenge.

Among the 11 RSV proteins, the non-structural proteins (NS1/2) cooperatively inhibit activation and nuclear translocation of interferon (IFN) regulatory factor 3 (Bossert *et al.*, 2003; Spann *et al.*, 2005), and mediate inhibition of cytokine production by proteasome-mediated degradation of the signal transducer and activator of transcription factor 2 (Elliott *et al.*, 2007; Lo *et al.*, 2005), whilst the surface proteins F and G are involved in attachment and entry, along with nucleolin (Tayyari *et al.*, 2011). The RSV G protein also interacts with Toll-like receptors (Kurt-Jones *et al.*, 2000; Murawski *et al.*, 2009; Oshansky *et al.*, 2009a), and negatively affects type I IFN (Moore *et al.*, 2008; Tripp *et al.*, 1999) and cytokine and chemokine expression (Tripp *et al.*, 2000a), in part by induction of suppressor of cytokine signalling (SOCS) proteins in normal human bronchoepithelial cells and mouse lung epithelial cells (Moore *et al.*, 2008; Oshansky *et al.*, 2009a). In addition, a highly conserved CX3C chemokine motif in the RSV G protein mimics fractalkine, modulating fractalkine-mediated immune responses (Tripp *et al.*, 2001).

RSV infection *in vitro* and *in vivo* induces early, middle and late host genome-wide gene transcription (Janssen *et al.*, 2007; Martínez *et al.*, 2007); however, these increases are not directly reflected in the host proteome (Munday *et al.*, 2010) and this is not completely understood. RSV infection causes G_1_/S arrest in A549 cells (Gibbs *et al.*, 2009) and HEp-2 cells (Mohapatra *et al.*, 2009), and a G_2_/M cell-cycle arrest in primary human bronchial epithelial cells via induction of transforming growth factor β1 and a reduction in p53, both *in vitro* and in *vivo*. RSV infection also induces stress granule formation via a protein kinase R-dependent pathway in HEp-2 cells, leading to increased virus replication in cytosolic viral inclusion bodies (Lindquist *et al.*, 2010, 2011). Components of stress granules are shared with processing bodies, which are sites for accumulation of 22 nt RNAs called microRNAs (miRNAs) (Fabian *et al.*, 2010; Selbach *et al.*, 2008), which regulate gene expression post-transcriptionally by binding to a complementary sequence in the 3′UTR of a target gene(s) via a ‘seed’ region (nt 2–7) in the miRNA, and cause transcript degradation or a block in translation (Fabian *et al.*, 2010; Selbach *et al.*, 2008). Consequently, miRNA deregulation linked to virus infection and replication can affect global gene expression. Recently, infection of normal tracheal epithelial cells with a recombinant GFP-expressing RSV (rgRSV) was shown to repress 24 miRNAs to varying extents (from −0.5-fold to −2.9-fold relative to mock-infected cells) and to induce two miRNAs (Othumpangat *et al.*, 2012). Of the miRNAs discovered, six were predicted to govern the neurotrophin nerve growth factor (NGF) gene, and miR-221 was shown to regulate NGF. As bronchial epithelia represent a mixed population of cells and miRNA expression can vary considerably among cell types, we investigated the deregulation of miRNA expression following RSV infection of A549 cells, a type II respiratory epithelial model. In this model, microarray data validated by quantitative real-time PCR (qPCR) showed that a different set of miRNAs (let-7f, miR-337, miR-520a, miR-24, miR-26b, miR-198 and miR-595) was deregulated following RSV infection. We determined that the RSV G protein modified let-7f expression and showed that let-7 miRNAs regulated several key host genes induced during RSV infection. Modulation of let-7f miRNA also regulated virus replication, a feature not attributable to the induction of antiviral cytokines alone. These studies suggest that RSV G protein-induced let-7 miRNA expression regulates host genes during RSV infection to modulate virus replication.

## Results

### RSV infection deregulates host miRNA expression

**Fig. 1.**
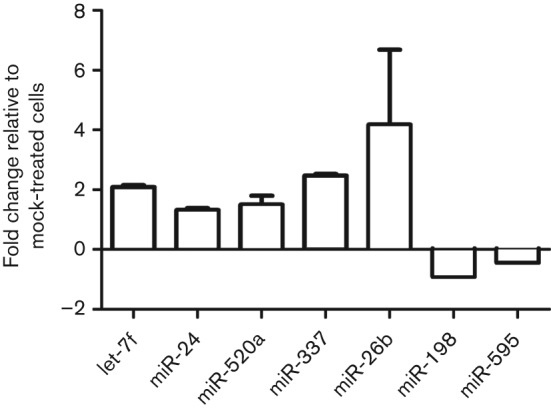
6340WT infection deregulates host miRNA expression. A549 cells were infected or mock infected with RSV 6340WT virus at an m.o.i. of 1 for 24 h. The data represent the mean qPCR fold change±sem of let-7f (let-7f), miR-337-3p (miR-337), miR-520a-5p (miR-520a), miR-24, miR-26b, miR-198 and miR-595 from three independent experiments relative to mock-infected cells, with values >1.0 considered to be upregulation and values below 1.0 considered to be downregulation.

To evaluate RSV deregulation of host miRNA expression, A549 cells were infected with recombinant wild-type RSV (6340WT; m.o.i. of 1) or were mock treated in triplicate, and expression of all known mature miRNAs was determined at 24 h post-infection (p.i.) by microarray analysis. The miRNAs let-7, let-7a, let-7f and miR-337 were significantly (*P*≤0.01) induced ≥1.5-fold among replicates, whilst miR-224 showed consistent repression of at least 1.5-fold among replicates (Table S1, available in JGV Online). The miRNAs miR-24, miR-26b, miR-29a, miR-320a and miR-520a-5p (miR-520a) were also induced ≥1.5-fold, whilst miR-198, miR-224 and miR-595 were repressed by at least 1.5-fold (Table S1). qPCR performed using miRNA-specific oligonucleotides validated approximate inductions of twofold for miRNAs let-7f and miR-337, 1.7-fold for miR-520a and miR-24 (Fig. 1) and fourfold for miR-26b (Fig. 1). Although multiple miRNAs were induced, we focused on let-7 miRNAs, as the induction of this family was consistent among replicates and let-7f showed the highest induction among various let-7 members.

### RSV G protein elicits miRNA expression

**Fig. 2.**
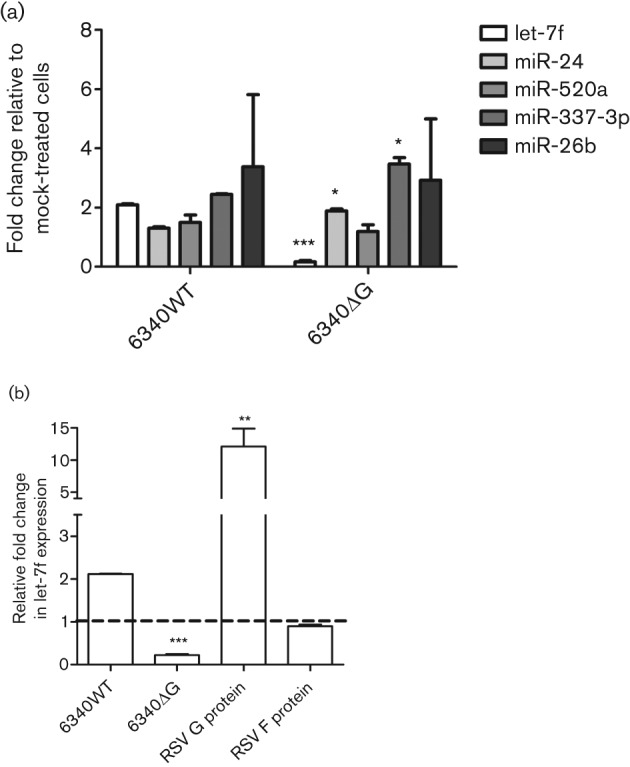
RSV G protein regulates miRNA expression during infection. (a) Expression of let-7f, miR-24, miR-520a, miR-26b and miR-337 was measured in A549 cells infected with 6340WT or 6340ΔG virus at 24 h p.i. (b) Expression of let-7f in cells treated with purified RSV G protein (1.0 µg ml^−1^) or RSV F protein (1.0 µg ml^−1^) and at 24 h after infection with 6340WT or 6340ΔG virus. Data represent mean fold changes in copy number±sem from three independent experiments relative to mock-infected cells. Statistical significance is indicated: ***P<0.001, **P<0.01, *P<0.05.

RSV G protein expression has been shown to modify cytokine and chemokine expression and to induce SOCS1- and SOCS3-negative regulation of type I IFNs (Moore *et al.*, 2008; Oshansky *et al.*, 2009a, b; Tripp *et al.*, 1999, 2000a, 2001). To determine whether RSV G protein affected expression of the validated miRNAs (Fig. 1), A549 cells were infected (m.o.i. of 1) with recombinant RSV (6340WT) or with a recombinant RSV mutant virus lacking the G gene (RSVΔG), and expression of let-7f, miR-337, miR-520a, miR-26b and miR-24 was determined at 24 h p.i. Both 6340WT and RSVΔG replicated to similar levels over the short period of infection; however, in the absence of the G protein gene (RSVΔG), expression of let-7f was significantly (*P*<0.001) lower, whilst levels of miR-337 and miR-24 were significantly (*P*<0.05) upregulated (Table 1 and Fig. 2a). Expression of miR-520a was decreased slightly in RSVΔG-infected cells, but this change was not statistically significant, and miR-26b expression remained unchanged compared with 6340WT infection. These results indicated that RSV G protein expression was associated with let-7f induction but repressed miR-24 and miR-337 expression. To confirm that RSV G protein induced let-7f (Fig. 2a), A549 cells were treated with purified RSV G protein (1.0 µg ml^−1^) (Oshansky *et al.*, 2009a), RSV F protein (1.0 µg ml^−1^) or infected (m.o.i. of 1) with 6340WT or 6340ΔG virus (Fig. 2b). A549 cells treated with purified RSV G protein showed a remarkable and significant (*P*<0.01) induction of let-7f relative to mock-treated cells, supporting a role for RSV G protein-mediated induction of miRNA let-7f. RSV F treatment did not change let-7f expression, confirming that RSV G is the major inducer for let-7f.

**Table 1.** Expression levels of miRNAs in A549 cells following infection with 6340WT or RSVΔG virus ns, Not significant. All experiments were carried out in duplicate.

miRNA	6340WT		RSVΔG		*P* value
	Mean	sem	Mean	sem	
let-7f	2.0900	0.060	0.1655	0.079	<0.0001
miR-24	1.310	0.070	1.890	0.084	0.0125
miR-337-3p	2.45	0.0141	3.47	0.296	0.013
miR-520a-5p	1.505	0.3464	1.196	0.316	ns
miR-26b	3.376	4.22866	2.923	3.60	ns

### let-7f regulates numerous host genes responding to RSV infection

**Fig. 3.**
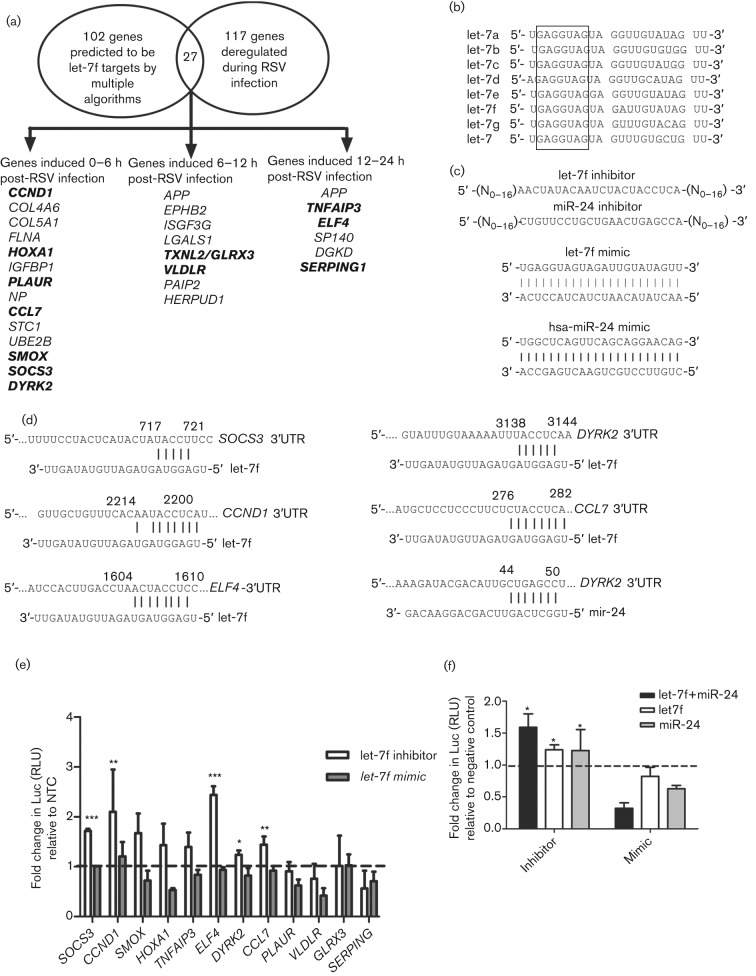
Luciferase (Luc)–UTR assays used to validate predicted let-7f gene targets. RSV G protein induced let-7f and other miRNAs regulate multiple genes during RSV infection. (a) Venn diagram depicting the overlap between predicted let-7f gene targets and genes deregulated during RSV infection. Genes that were examined further are shown in bold. SOCS3, Suppressor of cytokine signalling 3; CCND1, cyclin D1; SMOX, spermine oxidase; HOXA1, homeobox A1 transcription factor; TNFAIP3, tumour necrosis factor α-induced protein 3; ELF4, E74-like factor 4; DYRK2, dual-specificity tyrosine phosphorylation regulated kinase 2; CCL7, chemokine (C-C motif) ligand 7; PLAUR, plasminogen activator, urokinase receptor; VLDLR, very low density lipoprotein receptor; GLRX3, glutaredoxin 3; SERPING, serpin peptidase inhibitor, clade G. The dashed line indicates the baseline value. (b) Seed sequence (nt 2–8) conservation (boxed area) among miRNAs of the let-7 family. (c) Sequence of let-7f and miR-24 inhibitor and mimic sequences. The nature of chemical modifications (N_0–16_) on inhibitors and mimics are proprietary and not known. (d) Sequence alignment of various gene 3′UTRs and let-7f and miR-24. Numbers correspond to nucleotides in the 3′UTR. (e) let-7f regulates multiple genes during RSV infection. Luc–3′UTRs of putative let-7f targets were co-transfected into A549 cells with pSEAP2-Control (transfection control) and inhibitors or mimics for let-7f and/or miR-24. Data represent mean fold change±sem in Luc values [measured in relative light units (RLU)] from three independent experiments between inhibitor- and mimic-transfected cells relative to a non-target control (NTC) inhibitor or mimic. Statistical significance is indicated for all transfections represented in (e) and (f): ***P<0.001; **P<0.01; *P<0.05. (f) Cooperative activity of let-7f and miR-24 on DYRK2–Luc expression. A549 cells were transfected with DYRK2-pMLC plasmid and let-7f /miR-24 inhibitor/mimic alone or with DYRK2-pMLC plasmid and equimolar concentrations of let-7f+miR-24 inhibitor/mimic together with pSEAP2-Control plasmid as a transfection control. Data represent the fold change in Luc expression±sem from two independent experiments with the dashed line indicating the baseline value.

As RSV infection modified let-7 miRNA expression (Fig. 1), and RSV G protein expression considerably affected let-7f (Fig. 2), we focused on the role of let-7f in the host response. To identify let-7f targets regulated by base pairing between the miRNA ‘seed’ region and the 3′UTR of the gene (Friedman *et al.*, 2009), potential let-7f targets based on published genes linked to RSV infection and those predicted to be let-7f targets were mined using multiple computational algorithms (TargetScan, miRbase and PicTar). These potential targets were then mapped against a dataset of genes known to be deregulated at different times after RSV infection based on published microarray data (Fjaerli *et al.*, 2007; Janssen *et al.*, 2007; Martínez *et al.*, 2007; Wu *et al.*, 2011). From this analysis, 102 genes were identified as significant let-7f targets, 117 genes were found to be deregulated during RSV infection and 27 genes overlapped these two areas (Fig. 3a). The 27 genes were expressed at various time points (≤6 h, 6–12 h, and ≥12 h p.i.) after RSV infection of A549 cells, and were probably also regulated by other let-7 miRNAs, because of the 100 % identity in the let-7 ‘seed’ sequences (Fig. 3b) (Fjaerli *et al.*, 2007; Huang *et al.*, 2008; Martínez *et al.*, 2007). Regulation by let-7f of a subset of these genes was validated with let-7f inhibitors/mimics (Fig. 3c). Commercial let-7f and miR-24 inhibitor and mimics used in this study consistently prevented or increased the incorporation of the miRNA guide strand into the RNA-induced silencing complex (RISC) complex via proprietary design (Fig. 3c) (Vermeulen *et al.*, 2007). Different concentrations of let-7f inhibitors were tested, where 25 nM let-7f inhibitor reduced native let-7f levels by ≥85 % in 24 h and were not cytotoxic (Alamar Blue reduction assay; AbD SeroTec). Therefore, 25 nM was used in all transfection assays. It is important to note that, whilst the let-7f inhibitors were miRNA specific and were able to distinguish between different members of the let-7 family, let-7 mimics affected the native levels of all let-7 family members because the let-7 seed sites were identical across all let-7 miRNAs (Fig. 3b). Thus, for genuine let-7f targets, it would be expected that inhibitors and mimics would increase and decrease Luc expression, respectively, relative to non-targeting controls, although not to the same extent. Fold changes in Luc expression were calculated using the formula described in Methods. Moreover, based on findings from a previous study in human cells (Johnson *et al.*, 2007), the magnitude of differences in Luc expression would be expected to be modest compared with those of the controls.

The 3′UTRs for 12 (*SOCS3*, *CCND1*, *SMOX*, *HOXA1*, *TNFAIP3*, *ELF4*, *DYRK2*, *CCL7*, *PLAUR*, *VLDLR*, *GLRX3* and *SERPING1*) of the 27 genes representing early, middle and late times after RSV infection (Fjaerli *et al.*, 2007; Huang *et al.*, 2008; Martínez *et al.*, 2007) were cloned into a pMetLucControl (pMLC) plasmid that constitutively expresses secreted *Metridia longa* Luc from a cytomegalovirus promoter. Plasmid mixes of gene-specific *luc*–UTR constructs and transfection control plasmid [pSEAP2-Control, expressing secreted alkaline phosphatase (SEAP)] were co-transfected into A549 cells along with let-7f miRNA inhibitors or mimics (Dharmacon, Thermo Fisher), or with miRNA inhibitor negative control or mimic negative control that targeted *Caenorhabditis elegans* miR-67, and which have been validated not to affect the expression of any human gene (Dharmacon, Thermo Fisher).

Differences in Luc expression between let7-f inhibitor- and mimic-transfected cells were statistically (*P*<0.01) significant for five (*SOCS3*, *CCND1*, *ELF4*, *DYRK2* and *CCL7*) of the 12 genes examined relative to the negative controls (Fig. 3e). All these genes exhibited a strong match between the let-7f seed site and the 3′UTR of the target gene (Fig. 3d). Both let-7f and miR-24 were predicted to regulate the *DYRK2* gene (Fig. 3d). As miRNAs function as a molecular rheostat to fine-tune gene expression and may act cooperatively with other miRNAs (Asirvatham *et al.*, 2009), we investigated whether combined miRNA inhibition of let-7f and miR-24 further increased Luc expression. Accordingly, cells were transfected with DYRK2-pMLC plasmid and either transfected with let-7f or miR-24 inhibitor alone or co-transfected with DYRK2-pMLC plasmid and equimolar amounts of let-7f and miR-24 inhibitors or mimics. Concomitant inhibition of let-7f and miR-24 resulted in a significant (*P*<0.05) increase in Luc expression relative to cells transfected with let-7f or miR-24 inhibitor alone (Fig. 3f), suggesting that let-7f also acts cooperatively to regulate gene expression.

### let-7f regulates its targets via the RISC pathway

**Fig. 4.**
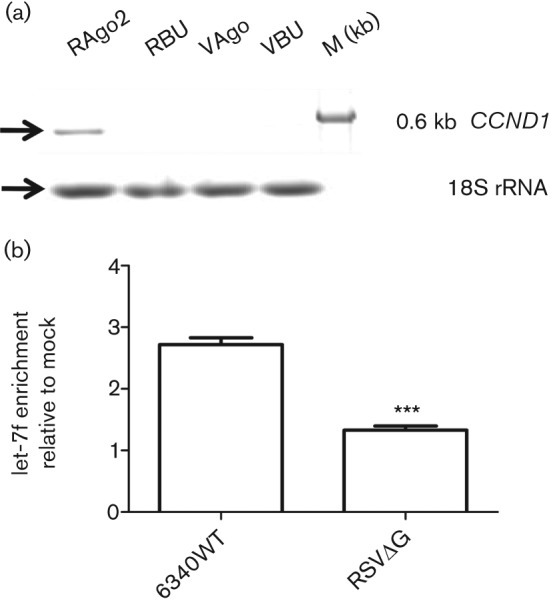
RISC complexes from RSV 6340WT-infected cells are enriched for *CCND1* and let-7f transcripts. (a) RISC-associated RNA from mock-, 6340WT- and 6340ΔG-infected cells were assayed for *CCND1* by PCR. *CCND1* UTR amplicons (0.6 kb) were amplified as described in Methods in two independent experiments. 18S rRNA was used as a loading control. RAgo, Anti-Ago2-precipitated RNA from RSV-infected cells; RBU, anti-BrdU-precipitated RNA from RSV-infected cells; VAgo, mock-infected Vero cell RNA precipitated with anti-Ago2; VBU, mock-infected Vero cell RNA precipitated anti-BrdU. (b) Enrichment of let-7f in RISC immunoprecipitated RNA from 6340WT- and RSVΔG-infected cells was assayed by qPCR and normalized to that of mock-infected cells from two independent experiments. Results are shown as means±sem, and Student’s *t*-test was used to measure the statistical significance of the data: ***P<0.001.

Gene transcripts regulated by miRNAs are processed by RISC complexes, which contain the Ago2 protein as a conserved core component (Fabian *et al.*, 2010). Precipitating RISC using an anti-Ago2 mAb has been shown to significantly enrich for miRNA-regulated transcripts (Dölken *et al.*, 2010). To validate let-7f-regulated transcripts, RISC-associated mRNA and target transcripts were precipitated from 6340WT-infected (m.o.i. of 1) or mock-infected cells using a mAb against Ago2 (a component of the RISC) or anti-bromodeoxyuridine (BrdU) control mAb. The *CCND1* gene, which is induced early during RSV infection (Martínez *et al.*, 2007), was enriched in anti-Ago2-precipitated RNA from RSV-infected cells but not in anti-BrdU-precipitated RNA from RSV-infected cells (Fig. 4a), or from similarly treated mock-infected Vero cell RNA precipitated with anti-Ago2 or anti-BrdU mAb (Fig. 4a). This showed that the *CCND1* transcript was associated with the RISC in RSV-infected cells, presumably for miRNA-mediated translational repression. As the RSV G protein induced let-7f (Fig. 2b), and let-7f regulated *CCND1* expression (Fig. 3e), it was expected that there would be differential enrichment of let-7f transcripts in RISC-associated RNA from 6340WT-infected cells compared with 6340ΔG-infected cells. qPCR analysis of RISC-associated mRNA precipitated from 6340WT-infected cells using anti-Ago2 mAb, but not using anti-BrdU mAb, showed an approximately threefold let-7f enrichment compared with RNA from 6340ΔG-infected cells (*P = *0.0005) (Fig. 4b). These results supported the findings showing RSV G protein induction of let-7f expression (Fig. 2b) and its involvement in regulating *CCND1* expression via the RISC pathway (Figs 3e and 4a).

### RSV replication is modulated by let-7f and miR-24

**Fig. 5.**
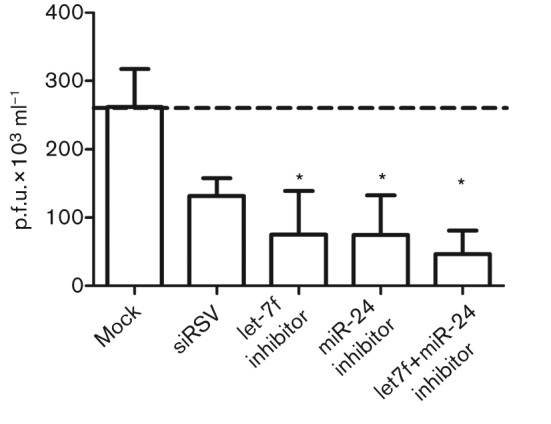
Modulation of miRNA levels deregulates virus replication. A549 cells were mock transfected or transfected in two independent experiments with inhibitors of let-7f and miR-24 separately and together (let-7f+miR-24), followed by infection with rgRSV at an m.o.i. of 0.5. The number of RSV p.f.u. was measured at day 3 p.i. using an anti-RSV F-based plaque assay relative to mock-infected cells. siRNA against the RSV N gene (siRSV) was used as a silencing control. The dashed line indicates basal p.f.u. levels in the mock-transfected control.

To determine whether let7-f affected RSV replication, A549 cells were transfected with let-7f miRNA inhibitor or mimic, or with controls, for 24 h and the cells were incubated for a further 48 h, assayed for cytotoxicity and subsequently infected with rgRSV expressing GFP at an m.o.i. of 0.5. Transfection with let-7f miRNA inhibitors, mimics or controls was not cytotoxic. rgRSV has been shown to replicate with a similar titre and time course as wild-type RSV in untreated A549 cells (Hallak *et al.*, 2000) At an m.o.i. of 0.5, rgRSV-infected A549 cells showed a peak GFP fluorescence at day 3 p.i. and hence were processed at this time point for RSV plaque assays on Vero E6 cells using an anti-RSV F-based plaque assay. The RSV plaque assays showed that inhibitor transfections reduced rgRSV plaque numbers, comparable to the results for positive-control siRNA (Fig. 5). A qPCR for RSV M gene copy numbers as well as GFP measurements also showed similar trends (data not shown). These data showed that miRNA inhibition can modulate RSV replication. As we did not observe statistically significant differences in antiviral cytokine expression after inhibitor/mimic transfection (unpublished observations), the effect on virus replication was probably due to a global deregulation of host gene expression. This is supported by a previous study showing that let-7 mimic transfections affected global gene expression profiles, deregulating 629 genes across multiple cellular pathways (Johnson *et al.*, 2007). We also analysed the RSV genome for potential seed sites for the above miRNAs using blast. The results (matrix = BLOSUM62, E value cut-off = 10.0) were filtered to identify hits in the miRNA seed site (nt 2–8). let-7f and miR-24 did not show any significant homology in the seed site with any region in the RSV genome in both sense and anti-sense orientations, ruling out a direct inhibition of virus replication by these miRNAs. This supports our hypothesis that modulation of virus replication in inhibitor-transfected cells is probably effected by modulating cellular pathways. These data suggest that RSV-modulated miRNAs have a pro-viral role and that modulation of these miRNAs affects virus replication by affecting multiple cellular pathways.

## Discussion

RSV is an important paediatric challenge, and understanding the miRNA regulated RSV–host interface is critical for vaccine development. As miRNA expression is regulated by multiple mechanisms such as Toll-like receptor recognition of pathogen-associated molecular patterns (Taganov *et al.*, 2006), extra- or intracellular signalling (O’Connell *et al.*, 2007; Taganov *et al.*, 2006), processing of IFN-stimulated gene transcripts (Berezikov *et al.*, 2007; Morlando *et al.*, 2008), direct viral induction of miRNA promoters (Taganov *et al.*, 2006) and as an off-target effect of viral inhibition of cellular processes, a central theme of this study was to determine the miRNAs that were deregulated following RSV infection in an established *in vitro* model of RSV infection, and to determine the effect of modulating these miRNAs on virus replication.

We identified a set of miRNAs that were deregulated (five induced and two repressed) during RSV infection of A549 cells where let-7f expression was induced most abundantly following RSV infection and was found to be regulated in part by RSV G protein. Treatment with purified RSV G protein enhanced let-7f expression and this was not observed following RSV F treatment. This is the first report of an RSV gene product regulating the expression of a host miRNA. let-7f showed maximum expression among differentially expressed let-7 miRNAs in A549 cells (copies per cell: let-7a, ~200; let-7b and let-7c, ~100; let-7e, ~50; let-7f, ~750; let-7g, ~80; let-7i, ~25; Johnson *et al.*, 2007), and our data are consistent with this study. The fold changes reported in this study are similar to a recently published study (Othumpangat *et al.*, 2012) that showed miR-221 deregulation in normal tracheal epithelial cells following rgRSV infection. miR-221 was postulated to be a major regulator of NGF, and miR-221 upregulation reduced NGF expression and virus replication.

To compare our findings with the above study, we performed an extensive analysis of miRNAs targeting NGF using ten different algorithms (DIANAmT, miRanda, miRDB, miRWalk, RNAhybrid, PICTAR4, PICTAR5, PITA, RNA22 and Targetscan) and failed to find any significant seed match between miR-221 and the NGF promoter and the 3′- or 5′UTR. Additionally, blast analysis of NGF mRNA (GenBank accession no. NM_002506.2) versus miR-221-5p (miRNA base accession no. MIMAT0004568) or miR-221-3p (miRNA base accession no. MIMAT0000278) failed to show any hits between the miR-221 seed site and the NGF-coding region, suggesting that the reported miR-221 regulation of NGF is an off-target effect of miR-221 transfection. In contrast, NGF treatment has been shown to induce miR-221/miR-222 via the ERK1/2-mediated pathway in culture (Terasawa *et al.*, 2009), and RSV infection induces NGF expression (Othumpangat *et al.*, 2009). miR-221 is a known negative regulator of the tumour suppressor genes *PTEN* (Zhang *et al.*, 2010b), *Bim* (Terasawa *et al.*, 2009) and *PUMA* (Zhang *et al.*, 2010a) and transcription factor *Foxo3a* (Hamada *et al.*, 2012). Hence, treatment with pre-miR-221 could be hypothesized to reduce the activity of these tumour suppressors, enhance apoptosis and reduce viral titres. It is important to note that Othumpangat *et al.* (2012) also identified miR-574 (repressed −0.5-fold relative to mock-infected cells) as a regulator of NGF, although its impact on virus replication was not studied. Differences between our findings and those above probably reflect the different cell types and viruses examined, as miRNA expression profiles vary considerably among cell types (Johnson *et al.*, 2007; Landgraf *et al.*, 2007).

let-7f gene targets were identified using a meta-analysis of computationally predicted let-7f targets and published microarray data on RSV-deregulated host genes. Of the genes predicted to be let-7f targets, 12 were tested using Luc–UTR assays and five genes (*CCND1*, *SOCS3*, *ELF4*, *DYRK2* and *CCL7*) showed modest but statistically significant differences following let-7f inhibitor and mimic treatment relative to non-targeting controls. The findings also showed that let-7f and *CCND1* transcripts co-localized in RISCs in RSV-infected but not in mock-infected cells using Ago2 immunoprecipitation, and were selectively enriched in 6340WT- versus RSVΔG-infected A549 cells, further supporting observations on the role of the RSV G protein. As let-7 miRNAs have 100 % sequence identity in their seed site, the results suggested that the let-7f target genes identified may also be regulated by other let-7 miRNAs. Inhibition of let-7f alone or in combination with miRNA miR-24 led to a significant reduction in rgRSV viral titres as measured by plaque assays. Lack of any significant homology between these miRNAs and the RSV genome and the negligible effects of these miRNAs on cytokine expression (unpublished observations) suggest that the observed reduction in viral titres is probably due to gene target modulation by let-7f.

CCND1 and ELF4 are important in cell-cycle regulation, affecting the G_1_/S phase transition, whilst ELF4 and DYRK2 inhibit p53-mediated induction of apoptosis (Maddika & Chen, 2009; Taira *et al.*, 2007; Taura *et al.*, 2011). CCND1, as a complex with CDK4/6, promotes G_1_/S phase transition, whilst DYRK2 regulates apoptosis via p53 phosphorylation (Taira *et al.*, 2007). ELF4 has been shown to be induced in RSV-infected cells at 12 h p.i., leading to expression of the E3 ubiquitin ligase Mdm2, which ubiquitinates p53 and targets it for proteasome-mediated degradation (Maddika & Chen, 2009). As previous studies have shown that RSV infection arrests cells in G_1_ (Johnson *et al.*, 2007; Mohapatra *et al.*, 2009), our findings of let-7 inhibition of CCND1, DYRK2 and ELF4 translation suggest that let-7-mediated gene regulation is one of the mechanisms employed by RSV (Groskreutz *et al.*, 2007; Mohapatra *et al.*, 2009).

The results of this study also showed that let-7f regulates *CCL7*/*MCP3* and *SOCS3*, two genes involved in the antiviral cytokine response. We have previously shown that RSV G protein-mediates inhibition of chemokine mRNA expression by bronchoalveolar leukocytes responding to RSV infection (Tripp *et al.*, 2000a) and induces interleukin-8 (IL-8) (Tripp *et al.*, 2000a), and in this study let-7f was shown to regulate ELF4, a known inducer of IL-8 (Hedvat *et al.*, 2004). Thus, RSV G protein expression is linked to let-7f deregulation and downstream modulation of IL-8 expression, which has been associated with RSV disease pathogenesis (Johnson & Graham, 2004). The RSV G protein is a well-documented immunomodulatory glycoprotein that is produced as both a membrane-bound and a soluble form (Roberts *et al.*, 1994) and is implicated in the induction of substance P, a neurokinin that mediates inflammation and enhanced pulmonary disease in RSV-infected BALB/c mice (Tripp *et al.*, 2000b). Importantly, RSV G protein expression has been linked to Th2-type cytokine skewing in the immune response to RSV infection in mice (Becker, 2006; Tripp *et al.*, 2000a, b; Varga *et al.*, 2000) and to inhibition of early chemokine mRNA expression (Tripp *et al.*, 2000a) via a highly conserved CX3C chemokine motif located in the central conserved cysteine-rich region (Harcourt *et al.*, 2006; Li *et al.*, 2006; Tripp *et al.*, 2001). The previous findings that RSV G protein inhibits type I IFNs through induction of *SOCS1* and *SOCS3* expression (Moore *et al.*, 2008; Oshansky *et al.*, 2009a) are consistent with the findings in this study showing RSV G protein induction of let-7f and governance of the *SOCS3* gene. It appears in this context that a consequence of RSV G protein expression is induction of *SOCS3*-negative regulation of type I IFNs, a process that would facilitate virus replication. However, this pro-viral function attributed to the RSV G protein appears to be balanced by the host response, where G protein also induces let-7f expression, which upregulated *SOCS3* expression.

An outcome of let-7f regulation of host genes seems to be delayed viral clearance. The data from Fig. 5 clearly showed that inhibition of let-7 and/or miR-24 affected virus replication significantly. These results suggest that host miRNAs may have a role in regulating virus replication similar to other RNA and DNA viruses (Jopling *et al.*, 2006; Lagos *et al.* 2010; Roberts *et al.*, 2011; Triboulet *et al.*, 2007), either by affecting cellular pathways or by directly regulating viral transcription and/or translation. Although we analysed host mRNAs in this study, we did not analyse the effect of these miRNAs on viral gene transcription or translation, and this could be an additional mechanism employed by the virus to control viral gene expression, similar to related viruses such as influenza. These findings provide a better understanding of the mechanisms that contribute to the host response to infection and disease pathogenesis, and move the field closer to the development of safe and effective RSV disease-intervention strategies. Further studies are needed to elucidate the spectra of potential mechanisms of miRNA activation associated with RSV infection in order to understand the kinetics of miRNA deregulation, the role of other viral proteins in miRNA deregulation and the global impact of these miRNAs on virus replication by other RNA viruses.

## Methods

### 

#### Cell culture and viruses.

Mycoplasma-free virus stocks of recombinant wild-type RSV strain A2 (6340WT) and RSV lacking only the G protein gene (RSVΔG; a kind gift of Dr Mark Peeples, Center for Vaccines & Immunity, The Research Institute at Nationwide Children’s Hospital, OH, USA) were expanded in Vero E6 cells (ATCC CCL-81) and maintained in Dulbecco’s modified essential medium (DMEM; Hyclone) supplemented with 5 % heat-inactivated FBS (Hyclone), as described previously (Oshansky *et al.*, 2009a). A549 cells (ATCC CCL-185) grown in DMEM supplemented with 5 % serum as above were used for all infections. A549 cells were infected at an m.o.i. of 0.5 or 1, as described previously (Oshansky *et al.*, 2009a).

#### qPCR for validation of miRNA microarray data.

miRNA DNA amplicons were generated from DNase-treated A549 RNA using an Ncode miRNA cDNA synthesis kit (Invitrogen) following the manufacturer’s instructions. Amplicons were quantified, and five tenfold cDNA dilutions in triplicate and equal amounts of 1 : 10-diluted cDNA from experimental samples in triplicate were used in a real-time qPCR assay on a Mx3000/Mx3005P instrument followed by dissociation curve analysis. Sequences of forward primers were based on mature miRNA sequences from miRbase (Table S2). Amplification was carried out using the following program: initial incubation at 52 °C for 2 min and 95 °C for 10 min, and 40 cycles of 15 s denaturation at 95 °C, 1 min annealing at 57 °C and 1 min extension at 68 °C, followed by dissociation curve analysis. Only standards/replicates showing an amplification efficiency between 90 and 110 % with an *R*^2^ value >0.985, and a slope of between −3.1 and −3.4 were used for copy number calculations. Fold changes in copy number were calculated and compared for statistical significance using Student’s *t*-test from duplicate experiments.

#### Computational analysis of miRNA targets.

Predicted host gene targets for the differentially expressed miRNAs were computationally mined from miRbase (Griffiths-Jones *et al.*, 2008), TargetScan (Lewis *et al.*, 2005) and PicTar (Krek *et al.*, 2005). Consensus target genes predicted by the three algorithms were compared with host genes identified previously to be affected by RSV infection (Martínez *et al.*, 2007) to narrow the number of genes that might be potential targets for RSV-deregulated miRNAs. The 3′UTR sequence of the genes of interest was amplified from A549 cDNA using oligonucleotides with a *Not*I site (GGCCGC) in the forward primer and an *Xba*I/*Spe*I site (TCTAGA/ACTAGT) in the reverse primer using LongAmp *Taq* (New England Biolabs) under the following conditions: initial denaturation at 94 °C for 30s and 30 cycles of 94 °C for 10 s, 55 °C for 30 s and 65 °C for 30s, with a final extension at 65 °C for 10 min. Correct-sized amplicons were cloned into a pMetLucControl (pMLC) plasmid (Clontech). The oligonucleotide sequences used for UTR cloning are shown in Table S3. Plasmids were verified by restriction digestion and sequencing.

#### Luc–UTR reporter plasmid design and Luc reporter assays.

All transfections were carried out in triplicate in at least three independent experiments. A549 cells (2×10^4^ per well) were transfected for 18 h using Lipofectamine 2000 with 200 ng gene-specific *luc*–UTR reporter plasmid (pMLC-UTR), 20 ng transfection control plasmid pSEAP2-Control (Clontech) and 25/50 nM specific or non-targeting miRNA mimics/inhibitor (NTC) to *C. elegans* miR-67 (Thermo Fisher) following the manufacturer’s instructions. The sequences of the miRNA inhibitors and mimics are given in Fig. 3(c). Luc and SEAP expression for each transfection was measured at 24 and 48 h post-transfection using a Ready-to-Glow kit (Clontech) following the manufacturer’s protocol. Fold changes in Luc expression were calculated using the formula: fold change in inhibitor or mimic = (Luc_test_/SEAP_test_)/(Luc_NTC_/SEAP_NTC_).

The data presented are means±sem from three independent experiments. Statistical significance was determined using Student’s *t*-test using GraphPad Prism version 5.0.

#### RISC immunoprecipitation assays.

Cell lysates were prepared at 24 h p.i. using cell lysis buffer [25 mM Tris/HCl (pH 7.5), 0.5 % NP-40, 150 mM KCl, 1 mM NaF, 2 mM EDTA, 0.5 mM DTT; all from Sigma] and a protease inhibitor tablet (Roche), and then used for RISC immunoprecipitation assays as described previously (Dölken *et al.*, 2010). Briefly, protein G–Sepharose beads (50 µl; Thermo Scientific) were incubated with rat anti-Ago2 hybridoma (a kind gift from Drs Juergen Haas, University of Edinburgh Medical School, Edinburgh, UK, and Gunther Meister, Universität Regensburg, Regensburg, Germany) or with BrdU mAb or FBS-free DMEM (controls) overnight at 4°C with mixing followed by two washes with cell lysis buffer to remove non-specifically bound material. Cell lysate from mock-treated or 6340WT-infected cells was added to the beads and incubated overnight at 4 °C to allow binding of the anti-Ago2–protein G complex with RISC complexes. The beads were washed five times in immunoprecipitation wash buffer [50 mM Tris/HCl (pH 7.5), 300 mM NaCl, 0.01 % NP-40, 5 mM MgCl_2_; all from Sigma], followed by one wash with ice-cold PBS to remove the detergent. RISC-associated RNA was extracted from the beads using Qiazol (Qiagen) and the RNA isolated following the miRNAeasy protocol described for qPCR.

#### miRNA transfections and virus replication and plaque assays.

All transfections were carried out as described above. Transfected cells were infected with mycoplasma-free rgRSV (m.o.i. of 0.5) for 2 h in serum-free DMEM, followed by a change to complete medium containing 5 % FBS. Cell lysates were prepared at 3 days p.i., sonicated and centrifuged at 300 ***g*** at 4 °C for 5 min. Tenfold dilutions of supernatant were made in serum-free DMEM on ice and 200 µl per well was added to 24-well plates containing 2×10^5^ Vero E6 cells per well in quadruplicate for 2 h followed by the addition of 1 ml 2 % carboxymethylcellulose. Plates were incubated for 6 days at 37 °C with 5 % CO_2_ and 95 % humidity, fixed with acetone : methanol (60 : 40) for 10 min at 4 °C and stained for RSV F protein using mAb 131-2A (produced in house). Plaques were detected with goat anti-mouse whole IgG coupled to alkaline phosphatase and developed using nitro blue tetrazolium (Thermo Fisher). Statistical analysis was carried out using a two-tailed Student’s *t*-test.
